# Using machine learning to develop preoperative model for lymph node metastasis in patients with bladder urothelial carcinoma

**DOI:** 10.1186/s12885-024-12467-4

**Published:** 2024-06-13

**Authors:** Junjie Ji, Tianwei Zhang, Ling Zhu, Yu Yao, Jingchang Mei, Lijiang Sun, Guiming Zhang

**Affiliations:** 1 Department of Urology, The Affiliated Hospital of Qingdao University, Qingdao, China; 2 Shandong Key Laboratory of Digital Medicine and Computer Assisted Surgery, The Affiliated Hospital of Qingdao University, Qingdao, China

**Keywords:** Bladder cancer, Lymph node metastasis, Bladder urothelial carcinoma, Machine learning, Risk

## Abstract

**Background:**

Lymph node metastasis (LNM) is associated with worse prognosis in bladder urothelial carcinoma (BUC) patients. This study aimed to develop and validate machine learning (ML) models to preoperatively predict LNM in BUC patients treated with radical cystectomy (RC).

**Methods:**

We retrospectively collected demographic, pathological, imaging, and laboratory information of BUC patients who underwent RC and bilateral lymphadenectomy in our institution. Patients were randomly categorized into training set and testing set. Five ML algorithms were utilized to establish prediction models. The performance of each model was assessed by the area under the receiver operating characteristic curve (AUC) and accuracy. Finally, we calculated the corresponding variable coefficients based on the optimal model to reveal the contribution of each variable to LNM.

**Results:**

A total of 524 and 131 BUC patients were finally enrolled into training set and testing set, respectively. We identified that the support vector machine (SVM) model had the best prediction ability with an AUC of 0.934 (95% confidence interval [CI]: 0.903–0.964) and accuracy of 0.916 in the training set, and an AUC of 0.855 (95%CI: 0.777–0.933) and accuracy of 0.809 in the testing set. The SVM model contained 14 predictors, and positive lymph node in imaging contributed the most to the prediction of LNM in BUC patients.

**Conclusions:**

We developed and validated the ML models to preoperatively predict LNM in BUC patients treated with RC, and identified that the SVM model with 14 variables had the best performance and high levels of clinical applicability.

**Supplementary Information:**

The online version contains supplementary material available at 10.1186/s12885-024-12467-4.

## Background

Bladder cancer (BC) is one of the most common urinary carcinomas, which was reported to be the tenth most common malignancy in both sexes and the sixth most common malignancy in men in 2020 worldwide [1]. As for patients with muscle-invasive bladder cancer (MIBC) and high-grade non-muscle‑invasive bladder cancer (NMIBC), treated with bladder-conserving therapies often result in early recurrence and progression. Therefore, radical cystectomy (RC) with pelvic lymph node dissection (PLND) is standardly recommended for these patients [2, 3].

Even if most patients treated with RC had negative surgical margins, approximately 50% patients had the possibility of recurrence, indicating the existence of extravesical tumor deposits at the time of surgery [4, 5]. Lymph node metastasis (LNM) is the most common site of BC metastases, which was reported to be ranging from 24 to 29% in patients receiving RC [4, 6]. It was reported that BC patients who had LNM had only 19% 5-year overall survival rate with RC treatment alone. Even if patients received RC combined with neoadjuvant or adjuvant chemotherapy, the 5-year overall survival rate was only around 30% [7]. Hence, preoperatively predicting LNM in patients with BC is necessary and beneficial.

Machine learning (ML) is one application field of artificial intelligence, which can automatically learn and improve the model performance without programming comparing with traditional methods [8]. Its algorithms can fit different configurations of data, assign weighting, and calculate the divinable power of each combination of variables in order to assess diagnostic and prognostic elements [9]. Several ML models which preoperatively predicted LNM in prostate cancer and renal cell carcinoma were established and validated, and some of which indicated the better performance compared with traditional logistic regression models [10, 11].

Over 90% of BC cases were pathologically diagnosed with bladder urothelial carcinoma (BUC). And previously we have established a traditional nomogram for predicting LNM in BUC, which was highly accurate, reliable, and clinically applicable in both internal validation and external validation [12]. However, there was no study aimed at preoperatively predicting LNM using ML in BC being reported. Therefore, we aimed to use ML algorithms to construct and validate a model for preoperatively predicting LNM in BUC using demographic information, imaging data, pathologic characteristics from transurethral resection of the bladder tumor (TURBT) specimens, and laboratory measurements.

## Methods

### Patient selection

This study was approved by the Medical Ethics Committee of the Affiliated Hospital of Qingdao University with the number of QYFYWZLL28026, and was carried out following the Declaration of Helsinki of the World Medical Association. We retrospectively collected the clinical data of patients who underwent RC and bilateral lymphadenectomy in the urology department of the Affiliated Hospital of Qingdao University between January 2013 and April 2022. We divided these patients into training set (80%) and testing set (20%) by stratified random sampling using the Stratified Shuffle-Split function in Python. LNM was defined as the confirmation of lymph node metastasis in the specimen from RC through pathology. Patients were excluded based on the following criteria: (a) age < 18 years; (b) without TURBT before RC or without muscle in TURBT; (c) patients with incomplete imaging examination data before RC; (d) tumor originated from sites other than the bladder; (e) patients with distant metastasis; (f) patients with incomplete laboratory measurements within a month before RC; (g) patients were diagnosed with non-urothelial carcinoma in pathology from RC; (h) patients receiving preoperative radiotherapy; (i) patients with severe or end-stage chronic kidney disease. This study complied with the principles of the Declaration of Helsinki and was conducted in accordance with the ethical standards of the medical ethics committee of our institution.

### Data collection

The following preoperative data of included patients were recorded: age, sex, body mass index (BMI), tumor grade of TURBT, papillary tumor presence of TURBT, urothelial variants of TURBT, muscle invasion of TURBT, infiltration of TURBT, hydronephrosis on imaging, extravesical invasion on imaging, positive LN on imaging, tumor size on imaging, neutrophil count, monocyte count, basophil count, eosinophil count, lymphocyte count, erythrocyte count, platelet count, hemoglobin, fibrinogen, urea nitrogen, creatinine, and albumin. We used the respective cell counts to calculate neutrophil to lymphocyte ratio (NLR), platelet to lymphocyte ratio (PLR), monocyte to lymphocyte ratio (MLR), and neutrophil to platelet ratio (NPR). Besides, the systemic immune-inflammation index (SII) was defined as multiplying the platelet count by the neutrophil count and dividing this value by the lymphocyte count.

The pathological characteristics (including tumor grade, papillary tumor presence, differentiation, muscle invasion, and infiltration) of the highest tumor grade or cancer stage were recorded when they received several rounds of TURBT. If the latest TURBT was performed over one month before RC, the pre-RC laboratory measurements were recorded. Otherwise, the measurements before TURBT were collected, which could reduce the impact of surgery on the results.

### Feature selection and model building

We conducted the univariate analysis for the recorded clinical variables to primarily determine potential preoperative risk factors for LNM in BUC. Secondly, spearman correlation analysis was performed to reduce collinearity among features. To reduce the risk of overfitting, the least absolute shrinkage and selection operator (LASSO) algorithm was applied to select features with non-zero coefficient values.

The prediction model of LNM in BUC patients after RC was established using five ML algorithms, including the support vector machine (SVM), light gradient boosting machine (LightGBM), eXtreme gradient boosting (XGBoost), random forest (RF) and extra-trees classifier. All patients were randomly categorized into training set (80%) and testing set (20%). The training set was used to establish the prediction models using five-fold cross-validation, whereas the testing set was used to validate the prediction models using the area under the curve (AUC) of the receiver operating characteristics (ROC) and corresponding 95% confidence intervals (95%CI). We considered the model with the highest AUC as the best model. We calculated the correlation coefficient between features and drew shapely additive explanation (SHAP) summary plot, which were used to visualize the relative importance ranking of each feature to the model predictions. Decision curve analysis (DCA) was performed to demonstrate net benefit for each risk threshold probability, as well as the clinical application value of the best model.

### Statistical analyses

The Stratified Shuffle-Split function was conducted in Python (version 3.7). Continuous variables with a normal distribution were described as means and standard deviations, continuous variables with an abnormal distribution were described as medians and interquartile ranges, and categorical variables were described as frequencies and proportions. Continuous variables with a normal distribution, continuous variables with an abnormal distribution, and categorical variables were univariately analyzed using Student’s t-test, Mann-Whitney U-test, and Chi-squared test, respectively. Univariate statistical analyses were performed using SPSS (version 24.0). Other statistical analyses, correlation analysis, and LASSO algorithm were implemented by importing the “scipy”, “numpy”, and “sklearn” packages in Python (version 3.7), and were performed using the “One-key AI” platform (http://www.medai.icu/), which was based on Python (version 3.7). The code used in this study was derived from: https://gitee.com/wangqingbaidu/OnekeyCompo. A bilateral P-value < 0.05 was considered as a measure of statistical significance.

## Results

### Patient characteristics

**Fig. 1:**
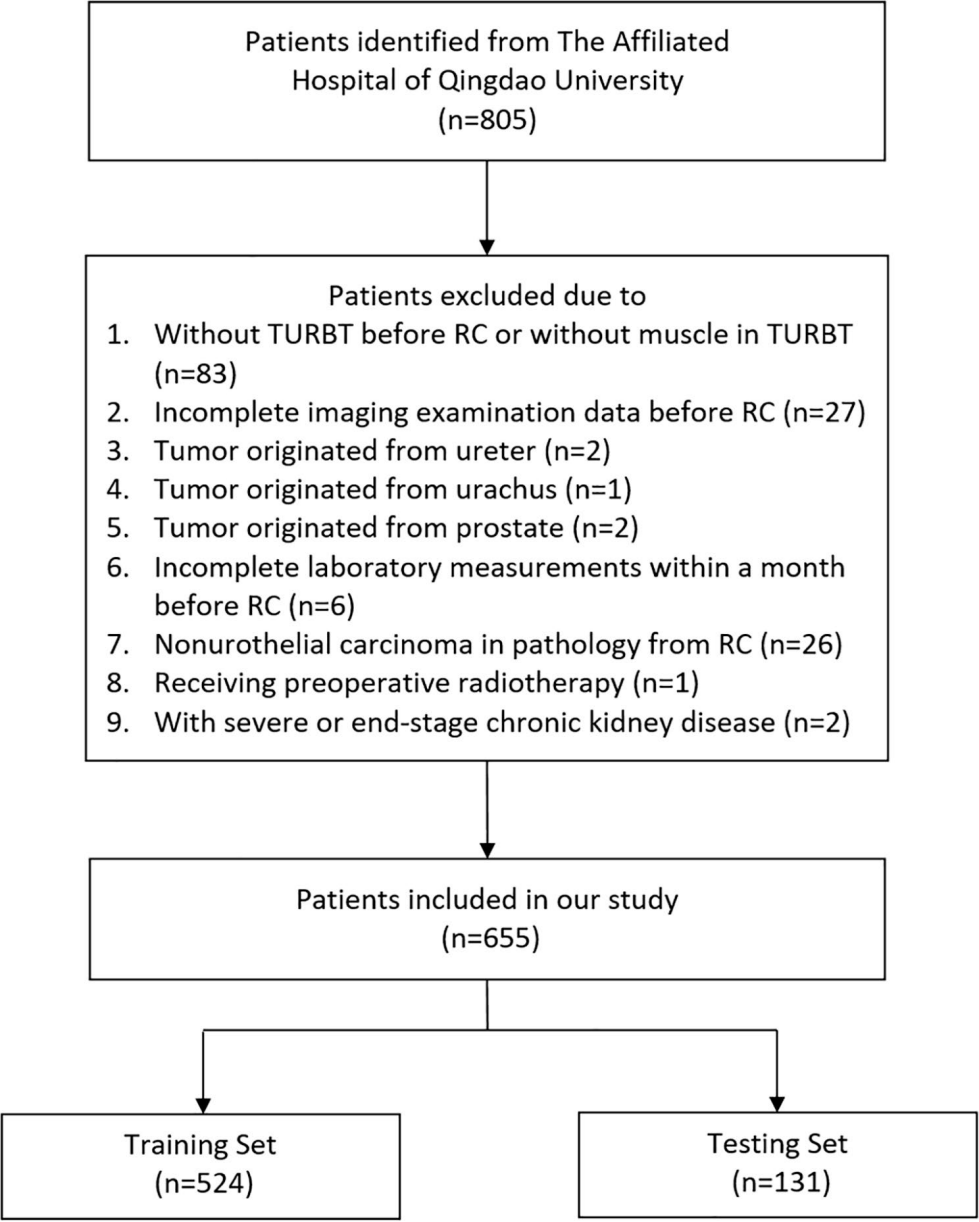
Flow chart of the process of patients’ selection

A total of 805 patients with BUC were potentially eligible from the Affiliated Hospital of Qingdao University between January 2013 and April 2022. After the selecting process, 655 patients were finally enrolled in our study, and 105 of which had LNM. The training set included 440 patients without LNM and 84 patients with LNM, while the testing set included 110 patients without LNM and 21 patients with LNM (Fig. 1). The baseline data of the included patient are shown in Table 1, which indicated that the grade, papillary, infiltration, hydronephrosis, extravesical invasion, positive lymph node, tumor size, neutrophil count, monocyte count, erythrocyte count, platelet count, hemoglobin, fibrinogen, creatinine, albumin, NLR, PLR, MLR, and SII were significantly different between patients with LNM and patients without LNM in univariate analyses.

**Table 1:** Baseline characteristics of the patients

Characteristics		LNM (+) (*n* = 105)	LNM (-) (*n* = 550)	*P* Value
**Demography**				
**Age**		67.0 (61.0–74.0)	66.0 (59.0–72.0)	0.060
**Sex**	male	93 (88.6%)	462 (84.0%)	0.233
	Female	12 (11.4%)	88 (16.0%)	
**BMI**		23.50 (20.80-26.05)	24.20 (22.00-26.20)	0.072
**Hypertension**	Yes	28 (26.7%)	161 (29.3%)	0.589
	No	77 (73.3%)	389 (70.7%)	
**Diabetes**	Yes	16 (15.2%)	61 (11.1%)	0.227
	No	89 (84.8%)	489 (88.9%)	
**Cardiovascular**	Yes	14 (13.3%)	62 (11.3%)	0.546
	No	91 (86.7%)	488 (88.7%)	
**Cerebrovascular**	Yes	5 (4.8%)	22 (4.0%)	0.927
	No	100 (95.2%)	528 (96.0%)	
**Pathology**				
**Grade**	High grade	102 (97.1%)	418 (76.0%)	< 0.001^***^
	Low grade	3 (2.9%)	132 (24.0%)	
**Papillary**	Yes	14 (13.3%)	219 (39.8%)	< 0.001^***^
	No	91 (86.7%)	331 (60.2%)	
**Urothelial** **Variants**	Yes	9 (8.6%)	44 (8.0%)	0.844
	No	96 (91.4%)	506 (92.0%)	
**Muscle Invasion**	Yes	15 (14.3%)	48 (8.7%)	0.077
	No	90 (85.7%)	502 (91.3%)	
**Infiltration**	Yes	81 (77.1%)	295 (53.6%)	< 0.001^***^
	No	24 (22.9%)	255 (46.4%)	
**Imaging**				
**Hydronephrosis**	Yes	55 (52.4%)	110 (20.0%)	< 0.001^***^
	No	50 (47.6%)	440 (80.0%)	
**Extravesical Invasion**	Yes	51 (48.6%)	86 (15.6%)	< 0.001^***^
	No	54 (51.4%)	464 (84.4%)	
**Positive LN**	Yes	36 (34.3%)	47 (8.5%)	< 0.001^***^
	No	69 (65.7%)	503 (91.5%)	
**Tumor Size (cm)**	≥ 4	64 (61.0%)	181 (32.9%)	< 0.001^***^
	< 4	41 (39.0%)	369 (67.1%)	
**Laboratory**				
**Neutrophil Count**		4.39 (3.29–5.93)	3.86 (3.07–5.08)	0.008^**^
**Monocyte Count**		0.53 (0.41–0.68)	0.48 (0.38–0.62)	0.019^*^
**Basophil Count**		0.03 (0.02–0.05)	0.03 (0.02–0.04)	0.795
**Eosinophil Count**		0.13 (0.07–0.24)	0.11 (0.06–0.18)	0.080
**Lymphocyte Count**		1.82 (1.35–2.17)	1.84 (1.43–2.25)	0.210
**Erythrocyte Count**		4.35 (3.87–4.70)	4.52 (4.18–4.84)	0.004^**^
**Platelet Count**		239 (199–293)	226 (188–263)	0.024^*^
**Hemoglobin**		136 (116–146)	139 (128–150)	0.001^**^
**Fibrinogen**		3.54 (3.00-3.97)	2.99 (2.56–3.55)	< 0.001^***^
**Urea Nitrogen**		6.44 (5.34–8.05)	6.22 (5.14–7.59)	0.217
**Creatinine**		89.70 (72.00-108.50)	80.04 (67.00-94.05)	< 0.001^***^
**Albumin**		39.45 ± 4.41	40.72 ± 4.41	0.007^**^
**NLR**		2.57 (1.93–3.41)	2.10 (1.56–2.92)	0.001^**^
**PLR**		134.00 (110.93-180.21)	123.87 (95.77-157.76)	0.014^*^
**MLR**		0.29 (0.24–0.41)	0.26 (0.20–0.35)	< 0.001^***^
**NPR**		0.018 (0.014–0.025)	0.017 (0.013–0.023)	0.162
**SII**		612.14 (400.63-882.79)	475.34 (329.76-696.77)	0.001^**^

LNM, lymph node metastasis; BMI, body mass index. *, *P* < 0.05; **, *P* < 0.01; ***, *P* < 0.001

LNM, lymph node metastasis; LN, lymph node; BMI, body mass index; NLR, neutrophil-to-lymphocyte ratio; PLR, platelet-to-lymphocyte ratio; MLR, monocyte-to-lymphocyte ratio; NPR, neutrophil-to-platelet ratio; SII, systemic immune inflammation index. *, *P* < 0.05; **, *P* < 0.01; ***, *P* < 0.001

The comparison of baseline characteristics between the training and testing sets with corresponding P values was shown in Table 2. LNM was not significantly different between training set and testing set (*p* = 1.000). All baseline characteristics between the training and testing sets were statistically insignificant except diabetes. Considering the rate of diabetes was not significantly different between patients with LNM or not (*p* = 0.227), the baseline characteristics between two sets were balanced. Besides, the baseline characteristics of the two sets by lymph node status were analyzed and shown in Supplementary Table 1.

**Table 2:** Comparison of baseline characteristics between the two sets

Characteristics		Training set (*n* = 524)	Testing set (*n* = 131)	*P* Value
**Demography**				
**Age**		66.0 (60.0–72.0)	67.0 (58.0–71.0)	0.871
**Sex**	male	449 (85.7%)	106 (80.9%)	0.174
	female	75 (14.3%)	25 (19.1%)	
**BMI**		24.00 (21.60–26.30)	24.09 (22.49-26.00)	0.992
**Hypertension**	yes	149 (28.4%)	40 (30.5%)	0.635
	no	375 (71.6%)	91 (69.5%)	
**Diabetes**	yes	52 (9.9%)	25 (19.1%)	0.004^**^
	no	472 (90.1%)	106 (80.9%)	
**Cardiovascular**	yes	56 (10.7%)	20 (15.3%)	0.143
	no	468 (89.3%)	111 (84.7%)	
**Cerebrovascular**	yes	20 (3.8%)	7 (5.3%)	0.432
	no	504 (96.2%)	124 (94.7%)	
**Pathology**				
**Grade**	high grade	413 (78.8%)	107 (81.7%)	0.469
	low grade	111 (21.2%)	24 (18.3%)	
**Papillary**	yes	190 (36.3%)	43 (32.8%)	0.463
	no	334 (63.7%)	88 (67.2%)	
**Urothelial** **Variants**	yes	45 (8.6%)	8 (6.1%)	0.352
	no	479 (91.4%)	123 (93.9%)	
**Muscle Invasion**	yes	49 (9.4%)	14 (10.7%)	0.643
	no	475 (90.6%)	117 (89.3%)	
**Infiltration**	yes	298 (56.9%)	78 (59.5%)	0.580
	no	226 (43.1%)	53 (40.5%)	
**Imaging**				
**Hydronephrosis**	yes	134 (25.6%)	31 (23.7%)	0.653
	no	390 (74.4%)	100 (76.3%)	
**Extravesical Invasion**	yes	109 (20.8%)	28 (21.4%)	0.885
	no	415 (79.2%)	103 (78.6%)	
**Positive LN**	yes	68 (13.0%)	15 (11.5%)	0.638
	no	456 (87.0%)	116 (88.5%)	
**Tumor Size (cm)**	≥ 4	195 (37.2%)	50 (38.2%)	0.840
	< 4	329 (62.8%)	81 (61.8%)	
**Laboratory**				
**Neutrophil Count**		3.90 (3.09–5.31)	3.95 (3.16–4.99)	0.816
**Monocyte Count**		0.50 (0.39–0.63)	0.47 (0.37–0.61)	0.276
**Basophil Count**		0.03 (0.02–0.04)	0.03 (0.02–0.04)	0.433
**Eosinophil Count**		0.12 (0.07–0.18)	0.10 (0.05–0.21)	0.342
**Lymphocyte Count**		1.84 (1.41–2.25)	1.82 (1.51–2.18)	0.823
**Erythrocyte Count**		4.51 (4.13–4.84)	4.48 (4.12–4.80)	0.787
**Platelet Count**		230 (190–265)	228 (187–278)	0.763
**Hemoglobin**		139 (126–150)	139 (127–147)	0.658
**Fibrinogen**		3.06 (2.62–3.68)	3.04 (2.59–3.68)	0.915
**Urea Nitrogen**		6.20 (5.16–7.60)	6.45 (5.20–8.17)	0.099
**Creatinine**		81.60 (68.00-96.80)	81.00 (66.20-98.13)	0.741
**Albumin**		40.44 ± 4.44	40.83 ± 4.39	0.365
**NLR**		2.21 (1.58–3.04)	2.04 (1.71–3.02)	0.758
**PLR**		125.46 (97.09-160.18)	130.53 (99.18-166.32)	0.481
**MLR**		0.27 (0.20–0.36)	0.25 (0.21–0.36)	0.552
**NPR**		0.018 (0.014–0.023)	0.017 (0.014–0.023)	0.501
**SII**		496.95 (338.77-722.38)	475.68 (334.93-761.85)	0.951
**Outcome**				
**LNM in pathology**	yes	84 (16.0%)	21 (16.0%)	1.000
	no	440 (84.0%)	110 (94.0%)	

BMI, body mass index. *, *P* < 0.05; **, *P* < 0.01; ***, *P* < 0.001

LNM, lymph node metastasis; LN, lymph node; NLR, neutrophil-to-lymphocyte ratio; PLR, platelet-to-lymphocyte ratio; MLR, monocyte-to-lymphocyte ratio; NPR, neutrophil-to-platelet ratio; SII, systemic immune inflammation index. *, *P* < 0.05; **, *P* < 0.01; ***, *P* < 0.001

### Features selection and model evaluation

**Fig. 2:**
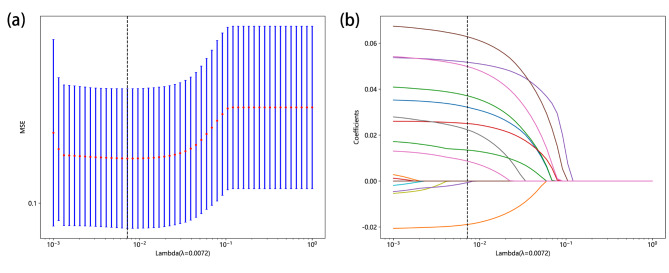
(**a**) The process of feature selection. We used the LASSO regression model with penalty parameter tuning conducted by fivefold cross validation according to minimum criteria. Selection of the tuning parameter (λ). Based on the minimum criteria, the vertical dotted line is plotted at the optimal value λ = 0.0072. (**b**) The vertical line was plotted with 14 selected features. LASSO, least absolute shrinkage and selection operator

We performed spearman correlation analysis and the lasso algorithm with fivefold cross-validation (Fig. 2a and b) to select predictors. 14 potential predictors of LNM after RC were ultimately determined (Supplementary Table 2), which were incorporated into the construction of the prediction model in our study.

**Fig. 3:**
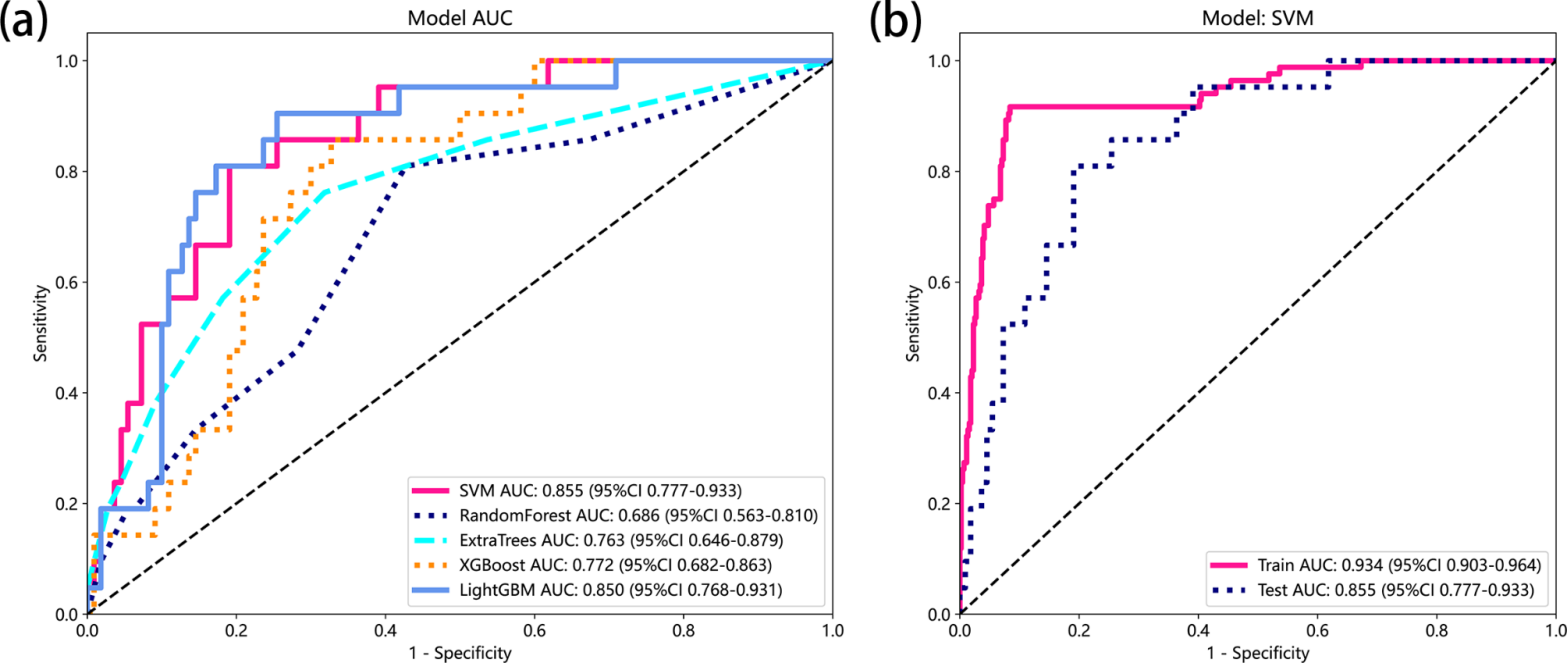
(**a**) Performance for machine learning models in the testing set based on the AUC of the ROC curve. (**b**) AUC and the ROC curve of SVM model in the training set and the testing set. AUC, area under the curve; ROC, receiver operating characteristics; SVM, support vector machine

Five machine learning algorithms utilizing the 14 selected factors as inputs were used to establish the prediction models in the training set, and the performance of the models was evaluated using the testing set and expressed by the AUC, accuracy, sensitivity, and specificity. The performance results of the prediction models in the training set and testing set were shown in Table 3. The receiver operating characteristics (ROC) and the area under the curve (AUC) for each different prediction models in the testing set were shown in Fig. 3a. The SVM model performed the best prediction ability with an AUC of 0.934 (95%CI: 0.903–0.964) and accuracy of 0.916 in the training set, and an AUC of 0.855 (95%CI: 0.777–0.933) and accuracy of 0.809 in the testing set (Fig. 3b). The RF model had the lowest AUC value of 0.686 (95% CI: 0.563–0.810) and accuracy of 0.611 in the testing set.

**Table 3:** Comparison of the performance of machine learning models in the training and testing set

Set	Model	Accuracy	AUC (95%CI)	Sensitivity	Specificity
**Training set**	SVM	0.916	0.934 (0.903–0.964)	0.917	0.916
	LightGBM	0.840	0.947 (0.928–0.966)	0.964	0.816
	Extra-Trees Classifier	1.000	1.000 (1.000–1.000)	1.000	1.000
	XGBoost	0.968	0.992 (0.983-1.000)	0.964	0.968
	RF	0.992	1.000 (0.999-1.000)	0.988	0.993
**Testing set**	SVM	0.809	0.855 (0.777–0.933)	0.810	0.809
	LightGBM	0.771	0.850 (0.768–0.931)	0.905	0.745
	Extra-Trees Classifier	0.695	0.763 (0.646–0.879)	0.762	0.682
	XGBoost	0.702	0.772 (0.682–0.863)	0.857	0.679
	RF	0.611	0.686 (0.563–0.810)	0.810	0.578

AUC, area under the curve; 95%CI, 95% confidence intervals; SVM, support vector machine; LightGBM, light gradient boosting machine; XGBoost, eXtreme gradient boosting; RF, random forest

### Importance of features of the best model

**Fig. 4:**
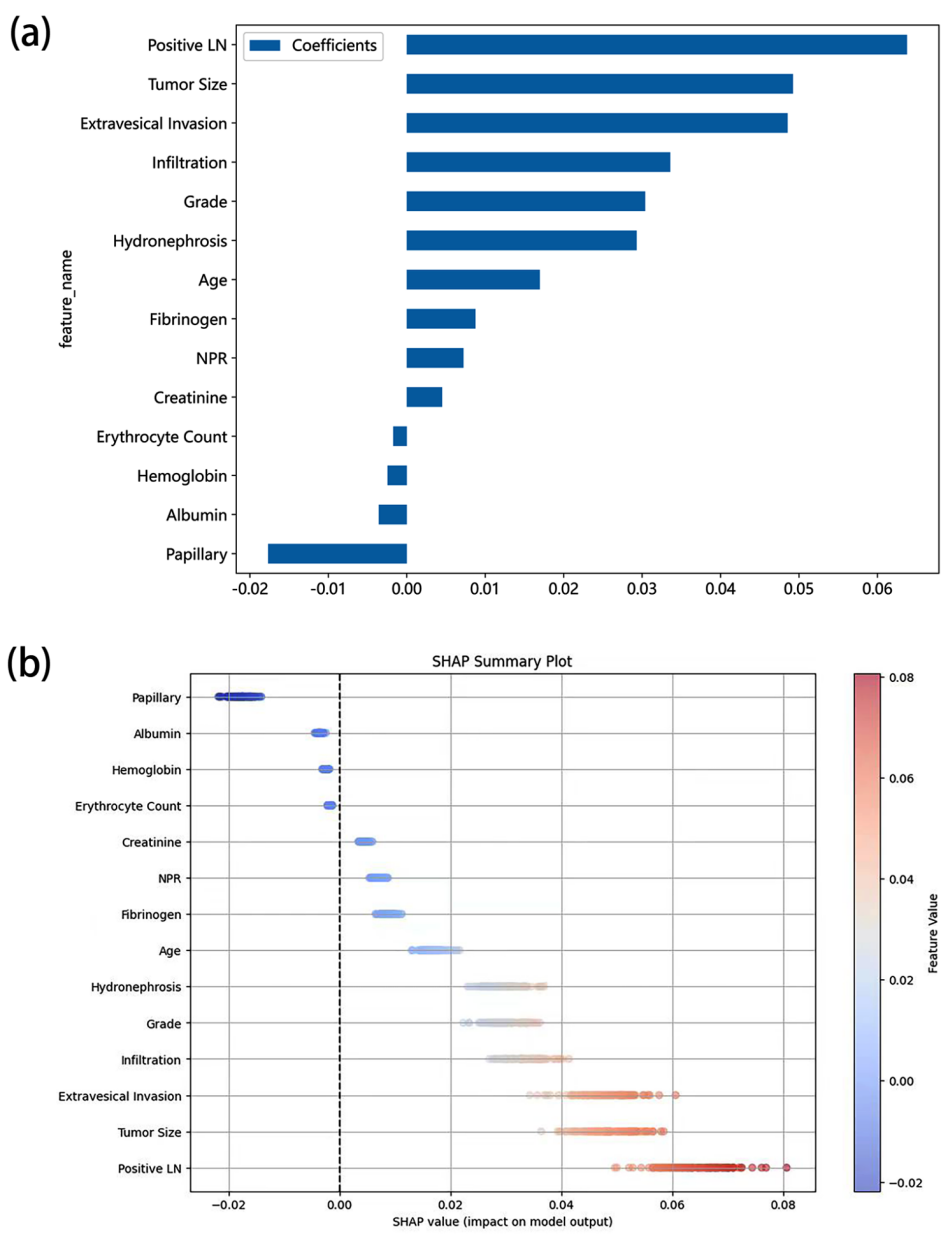
(**a**) Top 14 selected features and the corresponding variable coefficients of SVM model. Y-axis shows the top 14 variables, and X-axis shows their impact on the machine model. (**b**) SHAP summary plot of top 14 selected features of SVM model. SVM, support vector machine; LN, lymph node; NPR, neutrophil-to-platelet ratio; SHAP, shapely additive explanation

Coefficients were used to interpret the results of the best prediction model by evaluating the contribution of each variable to the prediction model. We focused on the SVM model since it was the best prediction model, and visualized these variables in Fig. 4a. Moreover, SHAP summary plot was also adopted to show the contribution of each predictor of LNM in the SVM model (Fig. 4b). The results revealed that positive lymph node in imaging contributed the most to the prediction of the outcome, followed by tumor size, extravesical invasion, infiltration, grade, hydronephrosis, papillary, age, fibrinogen, NPR, creatinine, albumin, hemoglobin, and erythrocyte count. Results of the DCA of SVM model in testing set showed that the model offered a clinical benefit at a threshold of between 0.10 and 0.50 (Supplementary Fig. 1).

## Discussion

RC plus bilateral PLND and neoadjuvant cisplatin-based combined chemotherapy are recognized as standard treatments of MIBC and some very high-risk NMIBC patients [2, 3]. However, the high possibility of concealed micro metastases resulted in the high recurrence rate of BUC after surgery [13]. Considering BUC patients with LNM were reported to have tumor tissue in LNs which were outside the region of standard PLND, the extended PLND were put up [14]. Even one study developed a nomogram aimed at LNM prediction in BC patients treated with extended PLND [15]. However, one prospective randomized trial demonstrated that extended LND showed no significant survival advantage over standard PLND [16]. Thus, preoperatively predicting LNM in BUC patients treated with RC is of high clinical value. Here, we developed and validated models for preoperatively predicting LNM in BUC using ML, and demonstrated that the SVM model performed the best prediction ability.

Several articles have extensively explored the independent predictors of LNM in BC after RC using various features. Two articles developed nomogram models containing gene signature. Cao et al. established epithelial-mesenchymal transition-LN signature containing 19 candidate genes and identified it as a predictor of LNM in BC [17]. Wu et al. also selected 5 LN-status-related mRNA and developed the five-mRNA-based classifier, which was also incorporated in the nomogram as an independent risk factor for LNM in BUC [18]. Besides, two studies used CT-based radiomics signature and MRI-based radiomics signature as independent variables to predict LNM in BC patients, respectively. And both CT-based radiomics signature contained nomogram and MRI-based radiomics signature contained nomogram showed good calibration and discrimination in the training and validation sets [19, 20]. However, the patients’ genomic and clinical features of gene-based nomograms were from online database such as The Cancer Genome Atlas, which could lead to the information selection bias and the restriction of the range of analyses. Although the radiomics features were selected from authors’ institution, the low sample size and the lack of external validation limited the validation and application of the nomogram models. Then, the genomic information and radiomics signature were difficult to collect and apply in clinical life, and finally restricted the clinical value of these models.

One study based on the Surveillance, Epidemiology, and End Results database identified age, tumor grade, tumor size, and tumor T stage as independent risk factors for LNM in BUC patients [21], which was similar to our selected predictors in the final ML model. Although the sample size was large, the AUC in training dataset was only 0.69, and the AUC in testing dataset was only 0.704, indicating the low accuracy of this model. Besides, another limitation was that the tumor grade in the database was from the pathology of RC, which could not be preoperatively collected. Ou et al. constructed a nomogram for predicting LNM in T1 high-grade BUC containing MLR and fibrinogen [22]. Another study also demonstrated that systemic inflammatory biomarker such as NLR was an independent risk factor for LNM in BUC [13]. Therefore, we selected information of laboratory measurements and calculated systemic inflammatory biomarkers. Although several articles reported that T stage was a risk factor of LNM in BC [13, 21], the staging accuracy of imaging tool such as CT was low. Therefore, we selected the “presence or absence of extravesical invasion” parameter instead of “T stage” to analyze. Although TURBT is typically recommended before RC and the resection specimen should contain bladder muscle tissue, the staging accuracy of TURBT is low, which was evidenced by the results that around 25–51% patients who were diagnosed with NMIBC in TURBT were upstaged to MIBC at RC [23–25]. The presence of lymphovascular invasion (LVI) was not accurately reported in our institution because immunohistochemistry is a nonessential tool in the diagnosis of BC [26]. Thus, we collected pathological information from TURBT excepted LVI. And finally, we developed the ML model based on preoperative demographic, pathological, imaging, and laboratory data, which were comprehensive and easily collected in clinical application.

We analyzed the relationship between positive LN on imaging and LNM in both training set and testing set using Chi-squared test, which was shown in Supplementary Table 1. Results indicated that positive LN was significantly different between patients with LNM and patients without LNM in both training set (*p* < 0.001) and testing set (*p* = 0.002). The accuracy of LN on imaging of diagnosing LNM was 0.821 and 0.832 in training set and testing set, respectively. However, the sensitivity was only 34.5% and 33.3% separately in training set and testing set, which was much lower than our model. Even though CT and MRI are most common imaging modalities in BUC, it was reported that both of them had limitation in the sensitivity of accessing LNM [3]. The sensitivity of diagnosing LNM in CT and MRI was only ranging from 14 to 30% [27, 28]. Even the most advanced imaging techniques such as PET-CT showed low sensitivity in predicting LNM [27, 29, 30]. Our machine learning model showed high accuracy, sensitivity, and specificity, which could aid clinical diagnosis. Positive LN still had the highest weight in our model (Fig. 4a and b), which might due to the high accuracy and specificity of this variable.

The precision medicine was commonly defined as the stratification of patients using clinical, lifestyle, genetic and further biomarker information with large-scale data [31]. ML is an accurate and new approach to facilely estimate individualized outcomes and bring better decision-making protocols with the availability of plenty of electronic patient clinical and genomic data at present [32]. Previously we had established a nomogram model for predicting LNM of BUC using multivariate traditional logistic regression. We identified tumor grade, infiltration, extravesical invasion, positive LN on imaging, tumor size, and serum creatinine levels as independent preoperative risk factors, while the AUC of 0.817 in training set and the AUC of 0.805 in testing set proved its accuracy and stability [12]. One study identified LNM related genes in prostate cancer using ML algorithm, and performed well in the validation process, indicating the excellent data handling capacity of ML methods [33]. Sabbagh et al. constructed a ML model to predict LNM in prostate cancer using standard clinicopathologic variables, and proved that ML model outperforms traditional tools by AUC and decision curve analysis [10]. The SVM model in our study containing 14 variables had AUCs of 0.934 and 0.855 in the training set and testing set, respectively, which were higher than the results in the traditional logistic regression model. Therefore, we concluded that the SVM model has better prediction performance in LNM of BUC patients than traditional model.

As far as we know, this is the first study to develop the ML model to preoperatively predict LNM in BUC patients using demographic, pathological, imaging, and laboratory data. The high AUCs in both training set and testing set demonstrated the high accuracy and discrimination ability of our model, and the large sample size guaranteed the stability of our results. Besides, the preoperative variables we selected were easily to get, facilitating the application of our model in clinical life.

Nevertheless, this study also had some limitations. First, the inaccurate data selection and the introduction of other potential confounders could not be eliminated due to the retrospective study design. Second, we only conducted the internal validation of all ML models and identified SVM model as the best model. The external validation with large sample size should be furtherly conducted. Third, several clinical trials and retrospective studies proved the survival benefit in BUC patients with neoadjuvant cisplatin-based chemotherapy [34–36]. Although neoadjuvant cisplatin-based chemotherapy has become the standard of care for cNOM0 patients with MIBC, we did not analyze the influence of neoadjuvant chemotherapy on LNM due to the lack of data. The relationship between neoadjuvant chemotherapy and LNM in patients with radical cystectomy should be explored with large sample size in the future. Finally, we only collected traditional pathological features of TURBT and traditional imaging data. One recently published study collected new pathological characteristics and radiomics features using deep learning algorithm and constructed the deep learning model to predict LNM in prostate cancer [37]. Thus, the micro variables of pathology and radiomics could be included and used to construct prediction model using deep learning in the future.

## Conclusions

We developed and validated the ML models to preoperatively predict LNM in BUC patients received RC, and identified that the SVM model with 14 variables had the best performance. The SVM model displayed high levels of accuracy and clinical applicability by internal validation.

### Electronic supplementary material

Below is the link to the electronic supplementary material.

Supplementary Material 1

Supplementary Material 2

Supplementary Material 3

## Data Availability

The datasets used and analyzed during the current study are available from the corresponding author on reasonable request. The code used in this study are available at: https://github.com/333JJJJ/Machine-Learning-Model.git.

## Abbreviations

LNM: Lymph node metastasis
BUC: Bladder urothelial carcinoma
ML: Machine learning
RC: Radical cystectomy
AUC: Area under the curve
SVM: Support vector machine
BC: Bladder cancer
MIBC: Muscle-invasive bladder cancer
NMIBC: Non-muscle‑invasive bladder cancer
PLND: Pelvic lymph node dissection
TURBT: Transurethral resection of the bladder tumor
BMI: Body mass index
NLR: Neutrophil to lymphocyte ratio
PLR: Platelet to lymphocyte ratio
MLR: Monocyte to lymphocyte ratio
NPR: Neutrophil to platelet ratio
SII: Systemic immune-inflammation index
LASSO: Least absolute shrinkage and selection operator
RF: Random forest
ROC: Receiver operating characteristics
SHAP: Shapely additive explanation
DCA: Decision curve analysis
95%CI: 95% confidence intervals
LVI: Lymphovascular invasion
