# Supplementary figures and images for: Using machine learning to develop preoperative model for lymph node metastasis in patients with bladder urothelial carcinoma
Source: BMC Cancer. 2024 Jun 13;24:725. doi: 10.1186/s12885-024-12467-4 (PMC11170799; doi:10.1186/s12885-024-12467-4)

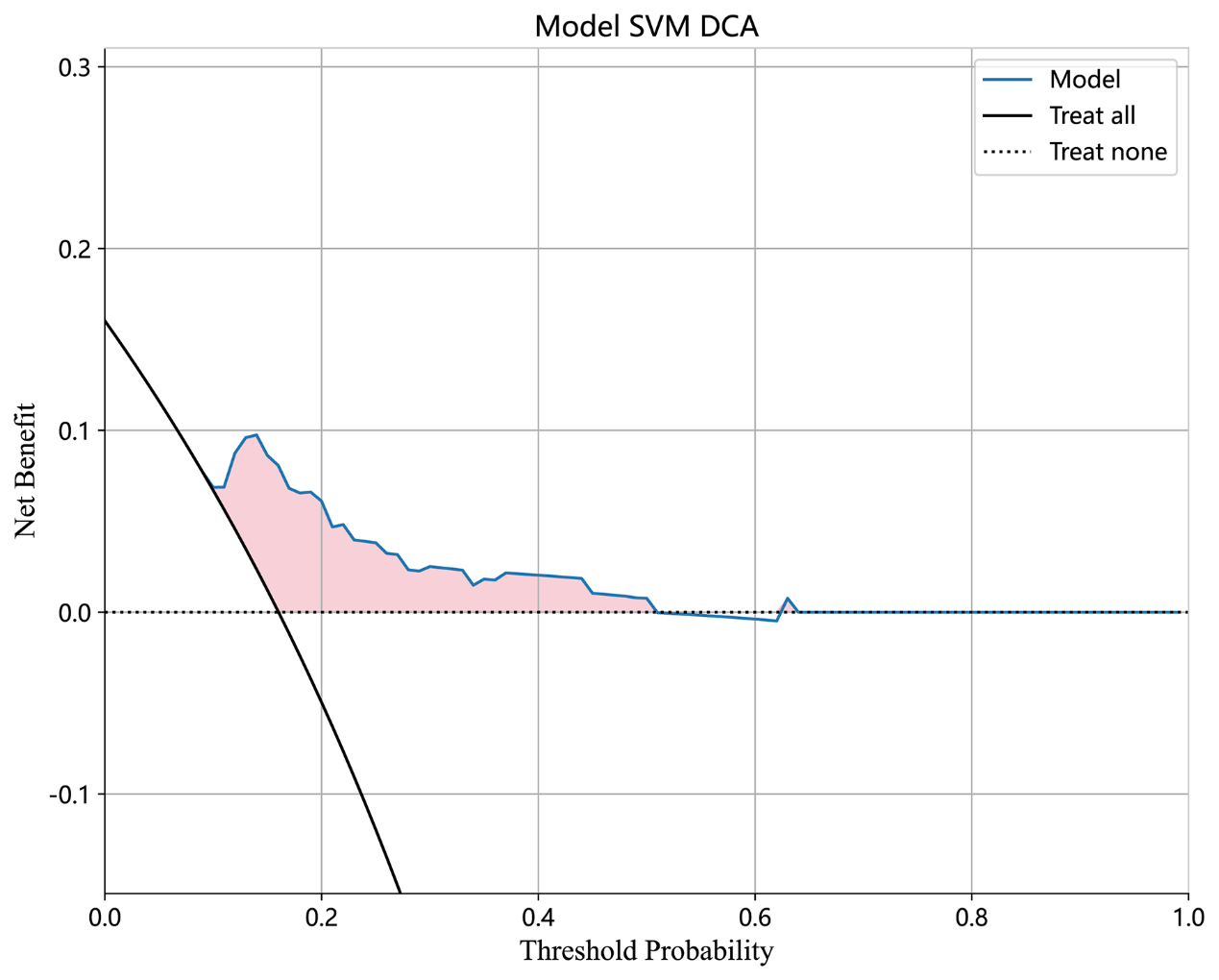


**Supplementary Fig. 1** Decision curve analysis of SVM model in testing set

Supplement: Supplementary file 1 — Supplementary Material 1 [file 12885_2024_12467_MOESM1_ESM.docx]
