# Supplementary material for: Using machine learning to develop preoperative model for lymph node metastasis in patients with bladder urothelial carcinoma
Source: BMC Cancer. 2024 Jun 13;24:725. doi: 10.1186/s12885-024-12467-4 (PMC11170799; doi:10.1186/s12885-024-12467-4)
Supplement: Supplementary file 2 — Supplementary Material 2 [file 12885_2024_12467_MOESM2_ESM.docx]

| **Supplementary Table 1** Characteristics of the two sets by lymph node status | | | | | | | |  |
| --- | --- | --- | --- | --- | --- | --- | --- | --- |
| **Characteristics** | **Training set (n=524)** | | |  | **Testing set (n=131)** | | |  |
|  | **LNM (-)**  **440** | **LNM (+)**  **84** | **P** |  | **LNM (-)**  **110** | **LNM (+)**  **21** | **P** | |
| **Demography** |  |  |  |  |  |  |  | |
| **Age** | 66.0 (59.0-72.0) | 66.0 (60.0-74.0) | 0.300 |  | 65.0 (57.8-71.0) | 70.0 (62.5-72.5) | 0.024^*^ | |
| **Sex** |  |  | 0.171 |  |  |  | 1.000 | |
| male | 373 (84.8%) | 76 (90.5%) |  |  | 89 (80.9%) | 17 (81.0%) |  | |
| female | 67 (15.2%) | 8 (9.5%) |  |  | 21 (19.1%) | 4 (19.0%) |  | |
| **BMI** | 24.18  (21.80-26.30) | 23.60  (21.03-26.00) | 0.251 |  | 24.20  (22.60-25.93) | 22.90  (19.50-26.25) | 0.082 | |
| **Hypertension** |  |  | 0.815 |  |  |  | 0.465 | |
| yes | 126 (28.6%) | 23 (27.4%) |  |  | 35 (31.8%) | 5 (23.8%) |  | |
| no | 314 (71.4%) | 61 (72.6%) |  |  | 75 (68.2%) | 16 (76.2%) |  | |
| **Diabetes** |  |  | 0.507 |  |  |  | 0.366 | |
| yes | 42 (9.5%) | 10 (11.9%) |  |  | 19 (17.3%) | 6 (28.6%) |  | |
| no | 398 (90.5%) | 74 (88.1%) |  |  | 91 (82.7%) | 15 (71.4%) |  | |
| **Cardiovascular** |  |  | 0.244 |  |  |  | 0.640 | |
| yes | 44 (10.0%) | 12 (14.3%) |  |  | 18 (16.4%) | 2 (9.5%) |  | |
| no | 396 (90.0%) | 72 (85.7%) |  |  | 92 (83.6%) | 19 (90.5%) |  | |
| **Cerebrovascular** |  |  | 0.421 |  |  |  | 0.510 | |
| yes | 15 (3.4%) | 5 (6.0%) |  |  | 7 (6.4%) | 0 (0.0%) |  | |
| no | 425 (96.6%) | 79 (94.0%) |  |  | 103 (93.6%) | 21 (100.0%) |  | |
| **Pathology** |  |  |  |  |  |  |  | |
| **Grade** |  |  | <0.001^***^ |  |  |  | 0.039^*^ | |
| high grade | 332 (75.5%) | 81 (96.4%) |  |  | 86 (78.2%) | 21 (100.0%) |  | |
| low grade | 108 (24.5%) | 3 (3.6%) |  |  | 24 (21.8%) | 0 (0.0%) |  | |
| LNM, lymph node metastasis; BMI, body mass index. *, P<0.05; **, P<0.01; ***, P<0.001. | | | | | | | | |

| **Supplementary Table 1** Continue 1 | | | | | | | |  |
| --- | --- | --- | --- | --- | --- | --- | --- | --- |
| **Characteristics** | **Training set (n=524)** | | |  | **Testing set (n=131)** | | |  |
|  | **LNM (-)**  **440** | **LNM (+)**  **84** | **P** |  | **LNM (-)**  **110** | **LNM (+)**  **21** | **P** | |
| **Papillary** |  |  | <0.001^***^ |  |  |  | 0.013^*^ | |
| yes | 178 (40.5%) | 12 (14.3%) |  |  | 41 (37.3%) | 2 (9.5%) |  | |
| no | 262 (59.5%) | 72 (85.7%) |  |  | 69 (62.7%) | 19 (90.5%) |  | |
| **Urothelial variants** |  |  | 0.928 |  |  |  | 0.829 | |
| yes | 38 (8.6%) | 7 (8.3%) |  |  | 6 (5.5%) | 2 (9.5%) |  | |
| no | 402 (91.4%) | 77 (91.7%) |  |  | 104 (94.5%) | 19 (90.5%) |  | |
| **Muscle Invasion** |  |  | 0.090 |  |  |  | 0.844 | |
| yes | 37 (8.4%) | 12 (14.3%) |  |  | 11 (10.0%) | 3 (14.3%) |  | |
| no | 403 (91.6%) | 72 (85.7%) |  |  | 99 (90.0%) | 18 (85.7%) |  | |
| **Infiltration** |  |  | <0.001^***^ |  |  |  | 0.029^*^ | |
| yes | 234 (53.2%) | 64 (76.2%) |  |  | 61 (55.5%) | 17 (81.0%) |  | |
| no | 206 (46.8%) | 20 (23.8%) |  |  | 49 (44.5%) | 4 (19.0%) |  | |
| **Imaging** |  |  |  |  |  |  |  | |
| **Hydronephrosis** |  |  | <0.001^***^ |  |  |  | <0.001^***^ | |
| yes | 91 (20.7%) | 43 (51.2%) |  |  | 19 (17.3%) | 12 (57.1%) |  | |
| no | 349 (79.3%) | 41 (48.8%) |  |  | 91 (82.7%) | 9 (42.9%) |  | |
| **Extravesical Invasion** |  |  | <0.001^***^ |  |  |  | <0.001^***^ | |
| yes | 71 (16.1%) | 38 (45.2%) |  |  | 15 (13.6%) | 13 (61.9%) |  | |
| no | 369 (83.9%) | 46 (54.8%) |  |  | 95 (86.4%) | 8 (38.1%) |  | |
| **Positive LN** |  |  | <0.001^***^ |  |  |  | 0.002^**^ | |
| yes | 39 (8.9%) | 29 (34.5%) |  |  | 8 (7.3%) | 7 (33.3%) |  | |
| no | 401 (91.1%) | 55 (65.5%) |  |  | 102 (92.7%) | 14 (66.7%) |  | |
| **Tumor Size (cm)** |  |  | <0.001^***^ |  |  |  | 0.001^**^ | |
| ≥4 | 146 (33.2%) | 49 (58.3%) |  |  | 35 (31.8%) | 15 (71.4%) |  | |
| <4 | 294 (66.8%) | 35 (41.7%) |  |  | 75 (68.2%) | 6 (28.6%) |  | |
| LNM, lymph node metastasis; LN, lymph node. *, P<0.05; **, P<0.01; ***, P<0.001. | | | | | | | | |

| **Supplementary Table 1** Continue 2 | | | | | | | |  |
| --- | --- | --- | --- | --- | --- | --- | --- | --- |
| **Characteristics** | **Training set (n=524)** | | |  | **Testing set (n=131)** | | |  |
|  | **LNM (-)**  **440** | **LNM (+)**  **84** | **P** |  | **LNM (-)**  **110** | **LNM (+)**  **21** | **P** | |
| **Laboratory** |  |  |  |  |  |  |  | |
| **Neutrophil Count** | 3.82 (3.07-5.17) | 4.54 (3.28-6.18) | 0.010^*^ |  | 3.93 (3.08-4.92) | 4.36 (3.28-5.20) | 0.469 | |
| **Monocyte Count** | 0.49 (0.38-0.62) | 0.54 (0.43-0.68) | 0.010^*^ |  | 0.47 (0.37-0.61) | 0.48 (0.38-0.65) | 0.935 | |
| **Basophil Count** | 0.03 (0.02-0.04) | 0.03 (0.02-0.04) | 0.794 |  | 0.03 (0.02-0.04) | 0.03 (0.01-0.05) | 0.972 | |
| **Eosinophil Count** | 0.12 (0.07-0.18) | 0.13 (0.07-0.22) | 0.252 |  | 0.10 (0.04-0.20) | 0.12 (0.08-0.31) | 0.091 | |
| **Lymphocyte Count** | 1.84 (1.42-2.29) | 1.83 (1.35-2.13) | 0.239 |  | 1.83 (1.52-2.18) | 1.71 (1.35-2.19) | 0.679 | |
| **Erythrocyte Count** | 4.52 (4.19-4.84) | 4.35 (3.85-4.71) | 0.012^*^ |  | 4.51 (4.15-4.85) | 4.35 (3.93-4.70) | 0.167 | |
| **Platelet Count** | 230 (189-263) | 230 (194-277) | 0.344 |  | 221 (186-260) | 295 (220-338) | 0.003^**^ | |
| **Hemoglobin** | 139 (128-151) | 137 (115-146) | 0.005^**^ |  | 141 (128-148) | 134 (116-140) | 0.028^*^ | |
| **Fibrinogen** | 3.01 (2.55-3.55) | 3.41 (2.93-4.01) | <0.001^***^ |  | 2.94 (2.57-3.55) | 3.79 (3.20-3.95) | <0.001^***^ | |
| **Urea Nitrogen** | 6.19 (5.11-7.57) | 6.28 (5.21-7.91) | 0.589 |  | 6.36 (5.20-7.81) | 6.82 (6.15-9.38) | 0.103 | |
| **Creatinine** | 80.00  (67.00-93.28) | 89.40  (72.28-107.80) | 0.001^**^ |  | 80.73  (66.00-95.80) | 93.89  (67.91-122.55) | 0.082 | |
| **Albumin** | 40.62±4.43 | 39.48±4.41 | 0.032^*^ |  | 41.12±4.32 | 39.33±4.53 | 0.087 | |
| **NLR** | 2.11 (1.55-2.89) | 2.59 (2.02-3.41) | 0.001^**^ |  | 2.06 (1.61-2.99) | 2.02 (1.86-3.26) | 0.437 | |
| **PLR** | 124.10  (95.80-157.80) | 129.71  (108.72-176.12) | 0.116 |  | 123.46  (94.82-158.28) | 153.08  (122.99-207.56) | 0.019^*^ | |
| **MLR** | 0.27 (0.20-0.35) | 0.31 (0.24-0.43) | <0.001^***^ |  | 0.25 (0.20-0.35) | 0.28 (0.23-0.39) | 0.340 | |
| **NPR** | 0.017  (0.013-0.023) | 0.020  (0.015-0.027) | 0.011^*^ |  | 0.018  (0.014-0.023) | 0.015  (0.013-0.017) | 0.047^*^ | |
| **SII** | 481.50  (330.00-693.92) | 592.46  (396.86-871.94) | 0.009^**^ |  | 455.62  (325.13-717.16) | 615.84  (451.03-969.58) | 0.026^*^ | |
| LNM, lymph node metastasis; NLR, neutrophil-to-lymphocyte ratio; PLR, platelet-to-lymphocyte ratio; MLR, monocyte-to-lymphocyte ratio; NPR, neutrophil-to-platelet ratio; SII, systemic immune inflammation index. *, P<0.05; **, P<0.01; ***, P<0.001. | | | | | | | | |
