# Supplementary material for: Using machine learning to develop preoperative model for lymph node metastasis in patients with bladder urothelial carcinoma
Source: BMC Cancer. 2024 Jun 13;24:725. doi: 10.1186/s12885-024-12467-4 (PMC11170799; doi:10.1186/s12885-024-12467-4)
Supplement: Supplementary file 3 — Supplementary Material 3 [file 12885_2024_12467_MOESM3_ESM.docx]

| **Supplementary Table 2** Coefficients for each selected characteristic | |
| --- | --- |
| **Characteristic** | **Coefficient** |
| Positive LN | 0.063770 |
| Tumor Size | 0.049237 |
| Extravesical Invasion | 0.048549 |
| Infiltration | 0.033582 |
| Grade | 0.030387 |
| Hydronephrosis | 0.029295 |
| Age | 0.016954 |
| Fibrinogen | 0.008747 |
| NPR | 0.007227 |
| Creatinine | 0.004504 |
| Erythrocyte Count | -0.001732 |
| Hemoglobin | -0.002456 |
| Albumin | -0.003566 |
| Papillary | -0.017680 |
| LN, lymph node; NPR, neutrophil-to-platelet ratio | |
